# Supplementary material for: Technosols Derived from Mining, Urban, and Agro-Industrial Waste for the Remediation of Metal(loid)-Polluted Soils: A Microcosm Assay
Source: Toxics. 2023 Oct 12;11(10):854. doi: 10.3390/toxics11100854 (PMC10610840; doi:10.3390/toxics11100854)
Supplement: Supplementary file 1 [file toxics-11-00854-s001.zip › toxics-2628356-supplementary.pdf]

Supplementary Material

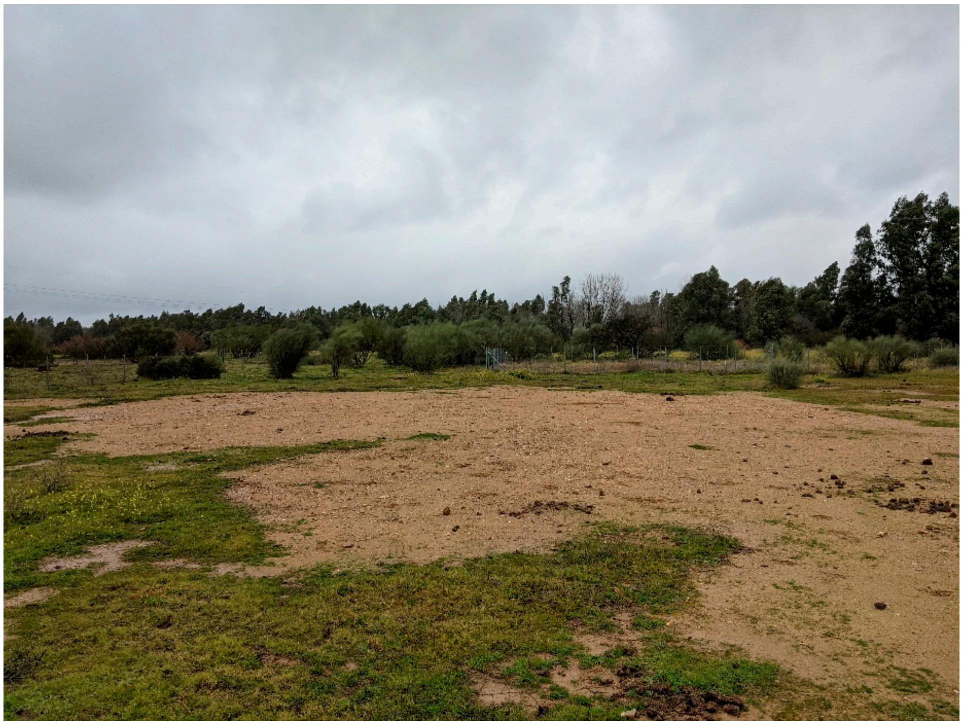

Figure S1. Residually polluted soils in the Guadiamar Green Corridor (Seville, Spain) more than 25 years after the Aznalcóllar disaster.

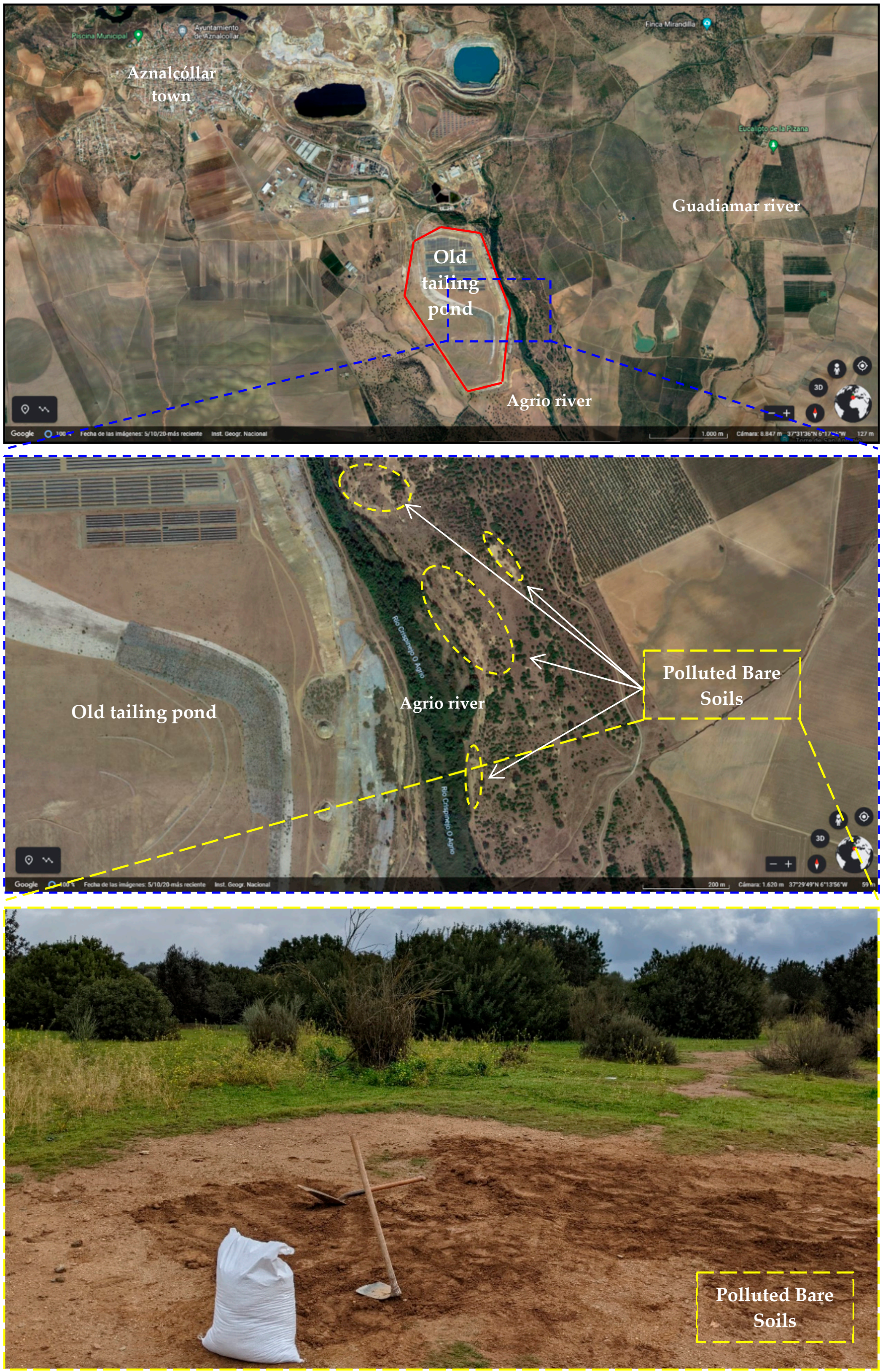

Figure S2. Identification of residually polluted soils, characterised by the absence of vegetation, in the proximity of the old tailings pond of the Aznalcóllar mine using satellite imagery.

Table S1. Main properties of the amendments used in the production of Technosols T1-T6 (IO, CW, MS, OL, WS, VC).

**Waste**	**pH** $_{(H_2O)\ 1:2.5}$	**EC** $_{1:5}$ (dS m^{-1})	**OC** (%)	**$CaCO_3$** (%)	**CEC** (cmol+ kg^{-1})	**Ca^{2+}** (cmol+ kg^{-1})	**Mg^{2+}** (cmol+ kg^{-1})	**Na^{+}** (cmol+ kg^{-1})	**K^{+}** (cmol+ kg^{-1})	**N_T** (%)	**C_T** (%)	**Fe_T** (%)	**P_A** (mg kg^{-1})	**BR** (µg CO_2 day^{-1} kg^{-1})
**IO**	7.27 ± 0.08	0.04 ± 0.01	n.d.	13.68 ± 0.22	6.34 ± 0.38	4.44 ± 0.39	n.d.	1.11 ± 0.03	0.79 ± 0.01	n.d.	1.55 ± 0.01	95.99 ± 0.88	n.d.	45.91 ± 4.42
**CW**	7.83 ± 0.04	2.81 ± 0.03	1.34 ± 0.03	93.12 ± 0.72	14.70 ± 0.50	2.75 ± 0.80	9.92 ± 0.67	1.15 ± 0.04	0.88 ± 0.01	0.07 ± 0.02	12.51 ± 0.03	0.28 ± <0.01	470.83 ± 6.16	124.05 ± 66.27
**MS**	8.27 ± 0.13	1.13 ± 0.08	0.16 ± 0.11	99.99 ± 0.01	9.34 ± 4.76	5.59 ± 4.60	1.44 ± 0.27	1.48 ± 0.13	0.84 ± 0.02	n.d	11.81 ± 0.05	0.17 ± 0.01	n.d.	29.46 ± 0.20
**OL**	8.63 ± 0.03	3.67 ± 1.86	28.08 ± 1.76	20.96 ± 1.25	90.72 ± 3.57	6.54 ± 2.00	2.22 ± 0.13	8.81 ± 0.03	73.15 ± 1.75	1.63 ± 0.28	30.60 ± 6.90	0.62 ± <0.01	318.70 ± 98.25	70.70 ± 32.73
**WS**	7.16 ± 0.02	10.13 ± 0.69	21.95 ± 2.91	10.77 ± 0.92	55.11 ± 5.81	24.94 ± 1.07	9.02 ± 0.71	5.67 ± 0.31	6.35 ± 0.19	3.13 ± 0.09	23.37 ± 0.35	2.01 ± 0.09	401.53 ± 14.07	13.95 ± 1.73
**VC**	7.35 ± 0.08	0.39 ± 0.07	10.50 ± 4.08	24.87 ± 1.30	35.83 ± 2.16	23.25 ± 2.00	8.36 ± 0.24	1.20 ± 0.02	3.02 ± 0.04	0.64 ± 0.02	12.44 ± 0.18	0.98 ± 0.13	226.94 ± 37.65	82.96 ± 9.36

IO – Iron oxyhydroxide-rich sludge, CW – Carbonated waste from peat extraction, MS –Marble cutting and polishing sludge, OL – Solid olive-mill by-product, WS – Composted sewage sludge, VC – Vermicompost from pruning and gardening, EC – Electric conductivity, OC – Organic carbon content, $CaCO_3$ - Calcium carbonate content, CEC Cation exchange capacity, N_T/C_T/Fe_T - Total concentrations of N, C and Fe, P_A – Assimilable phosphorus, BR – Basal respiration rate, n.d. - not detected. Extracted from [34].

Table S2. Retention effectiveness of metal(loid)s in simulated acid mine drainage of the amendments used in the production of Technosols T1-T6 (IO, CW, MS, OL, WS, VC).

	IO	CW	MS	OL	WS	VC
**As**	99.96 ± 0.01	99.74 ± 0.07	99.53 ± 0.27	81.72 ± 3.04	69.56 ± 6.98	99.67 ± 0.01
**Ba**		33.81 ± 36.49	40.27 ± 4.17			
**Be**	100.00 ± <0.01	100.00 ± <0.01	99.95 ± 0.09	62.80 ± 4.60	90.80 ± 2.04	99.74 ± 0.45
**Bi**	99.55 ± 0.24	97.93 ± 0.66	96.82 ± 1.43	92.16 ± 0.14	80.87 ± 8.10	99.76 ± 0.05
**Cd**	98.91 ± 0.04	99.68 ± 0.02	99.28 ± 0.02	94.85 ± 0.30	97.44 ± 0.30	98.54 ± 0.07
**Co**	98.80 ± 0.02	95.49 ± 0.22	64.25 ± 0.69	86.96 ± 0.47	83.57 ± 0.84	95.17 ± 0.59
**Cr**	100.00 ± <0.01	100.00 ± <0.01	100.00 ± <0.01	85.88 ± 1.63	83.52 ± 2.65	100.00 ± <0.01
**Cu**	99.93 ± 0.01	98.45 ± 0.01	99.46 ± 0.03	90.04 ± 0.60	92.49 ± 0.38	99.82 ± 0.01
**In**	100.00 ± <0.01	100.00 ± <0.01	99.94 ± 0.07	93.81 ± 0.58	91.86 ± 1.65	100.00 ± <0.01
**Mn**	98.02 ± 0.16	94.28 ± 0.30	78.43 ± 0.30	70.43 ± 1.03	73.12 ± 11.73	71.64 ± 2.50
**Mo**	3.45 ± 81.16		73.25 ± 2.85			
**Ni**	85.30 ± 0.16	74.74 ± 0.54	62.98 ± 0.76	47.79 ± 1.65	19.98 ± 17.38	87.42 ± 1.24
**Pb**	100.00 ± <0.01	100.00 ± <0.01	99.97 ± 0.05	90.90 ± 1.46	93.19 ± 4.65	99.93 ± 0.01
**Sb**	99.33 ± 0.03	98.13 ± 0.10	98.55 ± 0.06	84.31 ± 0.30	62.07 ± 6.33	98.27 ± 0.12
**Sc**	91.83 ± 0.09	95.55 ± 0.13	97.35 ± 0.43	71.76 ± 1.01	77.58 ± 4.79	90.34 ± 0.76
**Sn**	100.00 ± <0.01	100.00 ± <0.01	99.38 ± 0.54	87.11 ± 0.91	43.34 ± 22.57	99.70 ± 0.42
**Th**	98.95 ± 0.51	95.51 ± 1.03	95.88 ± 0.92	51.68 ± 1.85	61.90 ± 8.95	99.03 ± 0.21
**Tl**	95.86 ± 0.03	91.47 ± 0.16	60.10 ± 0.77	83.24 ± 0.72	90.98 ± 2.91	91.70 ± 0.02
**U**	80.20 ± 1.00		94.28 ± 0.17	71.68 ± 0.74	66.31 ± 7.12	94.28 ± 1.37
**V**	100.00 ± <0.01	100.00 ± <0.01	100.00 ± <0.01			100.00 ± <0.01
**Y**	99.93 ± 0.11	99.87 ± 0.22	99.92 ± 0.06	59.57 ± 2.14	79.26 ± 10.56	99.67 ± 0.06
**Zn**	99.22 ± 0.05	99.86 ± 0.01	93.63 ± 0.13	95.09 ± 0.36	95.17 ± 0.83	97.92 ± 0.19

IO – Iron oxyhydroxide-rich sludge, CW – Carbonated waste from peat extraction, MS –Marble cutting and polishing sludge, OL – Solid olive-mill by-product, WS – Composted sewage sludge, VC – Vermicompost from pruning and gardening. Adapted from [34].

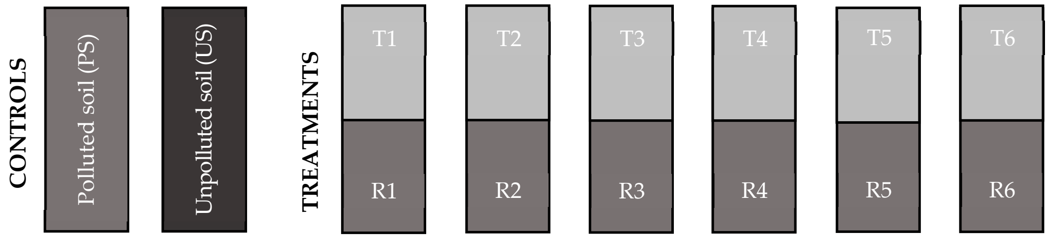

Figure S3. Diagram of the controls (PS and US) and rehabilitation treatments of the polluted soil with each of the Technosols (T1R1-T6R6).

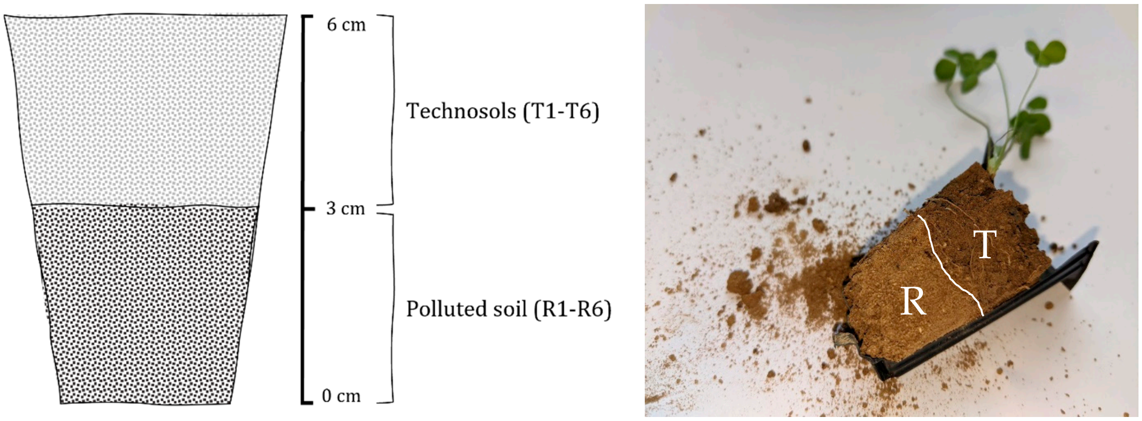

Figure S4. Scheme and close-up of the rehabilitation of polluted soils via surface application of Technosols (T1R1-T6R6).

Table S3. Results of the certified reference material of Loamy clay soil CRM 052-050 (RT-Corporation Limited, Salisbury, UK) analysed by X-ray fluorescence (XRF) with a portable NITON XL3t-980 GOLDD+ analyser (Thermo Fisher Scientific, Waltham, MA, Billerica, USA).

Element	Certified value	Prediction interval		Measured value	LOD
		Low	High		
**As**	14.60 (1.71)	10.90	18.30	15.53 (3.96)	7
**Cd**	35.60 (1.89)	31.60	39.60	41.15 (4.96)	12
**Cu**	44.20 (3.11)	37.60	50.80	48.51 (8.01)	13
**Fe**	12,400 (1,170)	9,870	14,900	11,187 (96)	N/A
**Pb**	82.60 (5.10)	71.80	93.40	92.62 (4.84)	8
**Sb**	20.10 (6.23)	6.13	34.20	30.25 (5.42)	20
**Zn**	89.00 (8.56)	70.90	107.00	94.41 (6.23)	10

Measured values are the mean of 6 replicates and standard error in parentheses. The limits of detection (LOD) are also shown. All values are given in mg kg^{-1}. N/A: Not applicable.

Table S4. Results of the certified reference material of Loamy clay soil CRM 052-050 (RT-Corporation Limited, Salisbury, UK) analysed by ICP-MS PerkinElmer® NexION™ 300D (Waltham, MA, USA).

Element	Certified value	Measured value	% (+/-)
**As**	14.60	12.80	-12
**Cd**	35.60	31.60	-11
**Cu**	44.20	32.70	-26
**Pb**	82.60	74.80	-9
**Sb**	20.10	23.73	+18
**Zn**	89.00	77.00	-13

Measured values are the mean of 6 replicates. All values are given in mg kg^{-1}.

Table S5. Principal component analysis (PCA) after Varimax rotation with Kaiser normalization including soil properties [pH, electrical conductivity (EC), organic carbon (OC) and $CaCO_3$ content, and total nitrogen and carbon (N_T, C_T)], total (T), water-soluble (W), and EDTA-bioavailable (E) concentrations of metal(loid)s, soil enzymatic activities (dehydrogenase, β-glucosidase, acid phosphatase, and cellulase), and endpoints calculated from *T. campestre* (survival and biomass) and *L. sativa* [seed germination (SG) and root elongation (RE)] toxicity bioassays. Only coefficients above ± 0.5 were included.

	Comp. 1	Comp. 2	Comp. 3
**pH**	0.834	-0.505	
**EC**		0.879	
**OC**			0.868
**$CaCO_3$**	0.892		
**N_T**			0.938
**C_T**	0.683		0.694
**As_T**		0.939	
**Cu_T**		0.649	0.655
**Pb_T**		0.943	
**Sb_T**		0.638	-0.654
**Zn_T**			0.684
**As_W**	0.836		0.534
**Cd_W**	-0.615	0.728	
**Cu_W**	-0.612	0.760	
**Pb_W**			0.799
**Sb_W**	0.954		0.502
**Zn_W**	-0.609	0.722	
**As_E**	0.748		
**Cd_E**	-0.881		
**Cu_E**	-0.662	0.658	
**Pb_E**	-0.555	-0.768	
**Sb_E**	0.945		
**Zn_E**	-0.825		
**Dehydrogenase**			0.873
**Β-glucosidase**		-0.760	-0.570
**Acid phosphatase**	0.517		
**Cellulase**		-0.518	
**Survival**		-0.520	-0.533
**Biomass**		-0.655	-0.533
**SG**			-0.613
**RE**	0.648	-0.682	
% ac. ex. var.	39.76	65.61	81.44
